# The *Interleukin-6* gene variants may protect against SARS-CoV-2 infection and the severity of COVID-19: a case-control study in a Moroccan population

**DOI:** 10.1186/s12920-024-01911-w

**Published:** 2024-05-23

**Authors:** Rachid Noureddine, Hanâ Baba, Safaa Aqillouch, Karima Abounouh, Oumaima Laazaazia, Mohcine Elmessaoudi-Idrissi, Fatima Zohra Bahmani, Ikram Allah Tanouti, Ahd Ouladlahsen, M’hammed Sarih, Hind Dehbi, Sayeh Ezzikouri

**Affiliations:** 1 Virology Unit, Viral Hepatitis Laboratory, Institut Pasteur du Maroc, 1 Place Louis Pasteur, Casablanca, 20360 Maroc; 2 Laboratory of Genetics and Molecular Pathology, Medical School, University Hassan II, Casablanca, Maroc; 3 Laboratoire Morizgo d’analyses médicales, Casablanca, Maroc; 4 Service des maladies Infectieuses, CHU Ibn Rochd, Casablanca, Maroc; 5 Service de Parasitologie et des Maladies Vectorielles, Institut Pasteur du Maroc, Casablanca, Morocco

**Keywords:** COVID-19, *Il-6*, Susceptibility, Promoter polymorphisms, Proinflammatory cytokine, Genotype, Haplotypes

## Abstract

The symptoms of SARS-CoV-2 infection vary widely, ranging from asymptomatic cases to severe forms marked by acute respiratory distress syndrome, multi-organ damage, and fatalities. Studies indicate a correlation between specific genes and susceptibility to SARS-CoV-2 infection and disease severity, particularly involving variants in genes linked to inflammation and immune responses. The objective of this study is to investigate the association between rs1800795 (− 174 G > C) and rs1800797 (− 597 A > G) variants in the *interleukin-6 (IL-6)* promoter region and susceptibility to SARS-CoV-2 infection. Additionally, we aim to explore their correlation with COVID-19 severity in a Moroccan population. In this case-control study, we enrolled 270 unvaccinated COVID-19 patients, consisting of 132 with severe COVID-19 and 138 with asymptomatic-moderate COVID-19. Additionally, we included 339 SARS-CoV-2-negative group. Genotyping of rs1800795 and rs1800797 polymorphisms of the *IL-6* gene was performed using predesigned TaqMan SNP genotyping. The median age of SARS-CoV-2-negative controls was 50 years, while severe COVID-19 cases exhibited a median age of 61 years. Additionally, individuals with asymptomatic to moderate COVID-19 had a median age of 36 years. We observed a significant age difference between severe and mild COVID-19 patients (*p* < 0.0001), and an association was noted between gender and the severity of COVID-19 (*p* = 0.011). The allele and genotype frequencies of the *IL-6* − 597G > A and − 174G > C variants did not show significant associations with susceptibility to SARS-CoV-2 infection (*p* > 0.05). However, further analysis revealed that the linkage disequilibrium between rs1800797 and rs1800795 indicated that individuals with the GC* haplotype (OR = 0.04, 95% CI 0.01–0.30, *p* = 0.001) and AG* haplotype (OR = 0.11, 95% CI 0.03–0.46, *p* = 0.002) were significantly associated with protection against SARS-CoV-2 infection. Moreover, in the overdominant model, the *IL-6* − 174 G/C genotype was found to be protective against the development of severe disease compared to those with the G/G-C/C genotypes (*p* = 0.03; OR = 0.41, 95% CI 0.18–0.96). However, correlations between complete blood count markers, hematological markers, D-dimer, C-reactive protein, and ferritin levels according to − 597 A > G and − 174G > C genotypes showed no significant differences (all *p* > 0.05). Our findings provide valuable insights into the pathogenesis of COVID-19, suggesting that genetic variations at the *IL-6* gene may contribute to the susceptibility to severe SARS-CoV-2 infection within the Moroccan population.

**Supplementary Information:**

The online version contains supplementary material available at 10.1186/s12920-024-01911-w.

## Introduction

The coronavirus disease 2019 (COVID-19) pandemic caused by the severe acute respiratory syndrome coronavirus-2 (SARS-CoV-2) rapidly emerged as a global pandemic round the world. As of December 24, 2023, there have been 773,119,173 million confirmed cases and more than 6,990,067 million deaths worldwide due to COVID-19, posing an unprecedented challenge to humanity (https://data.who.int/dashboards/covid19/deaths?n=c).

The clinical picture of patients infected with SARS-CoV-2 ranges from asymptomatic to severe courses of COVID-19 with acute respiratory distress syndrome (ARDS), multiorgan involvement and death. The initial symptoms of COVID-19 mainly include fever, cough, headache, fatigue, breathing difficulties, anosmia and ageusia [1]. Previous studies suggested that SARS-CoV-2 infection triggers an immune dysregulation known as a cytokine storm (CS) characterized by the high-level of proinflammatory cytokines and other inflammatory markers and has been suggested to be associated with COVID-19 severity [2, 3]. This hyperinflammatory syndrome triggers the severe inflammatory response observed in SARS-CoV-2 infection and causes coagulopathies, oxidative stress, organ damage, and death [4]. Interleukin 6 (IL-6) is one of the most widely recognised cytokine markers of inflammation, providing clinicians with valuable information for the early identification of patients with severe COVID-19 during the course of the disease [5].

SARS-CoV-2 triggers the activation of pathogenic Th1 cells, leading to the secretion of proinflammatory cytokines, including granulocyte-macrophage colony-stimulating factor (GM-CSF) and IL-6. GM-CSF, in turn, stimulates CD14 + CD16 + inflammatory monocytes to generate substantial amounts of IL-6, tumor necrosis factor-α (TNF-α), and other cytokines [4]. Furthermore, IL-6 binds to the soluble form of the IL-6 receptor (sIL-6R) and the membrane-bound IL-6 receptor (mIL-6R) through gp130, inducing various proinflammatory cytokines and chemokines. This pleiotropic effect extends to acquired and innate immune cells, respectively, ultimately leading to cytokine storms [4, 6]. Interestingly, a previous study found that tocilizumab (TCZ), an IL-6 receptor antagonist capable of inhibiting cytokine storms by blocking the IL-6 signal transduction pathway, could decrease the likelihood of progression to the composite outcome of mechanical ventilation or death. However, it did not demonstrate an improvement in overall survival [7].

The human *IL-6* gene is located on chromosome 7p21 and harbour many single-nucleotide polymorphisms (SNPs) in the coding and non-coding regions. The *IL-6* gene possesses a 303 bp promoter, where the adenine (A) to guanine (G) substitution at the − 597 position (− 597 A > G; rs1800797) and the guanine (G) to cytosine (C) replacement at the − 174 position (− 174G/C; rs1800795) are notable [8]. Indeed, previous data have highlighted that genetic polymorphisms in the promoter region exert an influence on IL-6 transcription [9].

Genetic factors within the host organism can play a significant role in the development and severity of SARS-CoV-2 infection. This includes immune-related genes, which can ultimately influence the clinical outcome of the disease [1, 10]. Investigating the role of genetic factors in the susceptibility and severity of COVID-19 can enhance our understanding of the pathogenetic mechanisms underlying complications and fatalities associated with this disease. It has been flagged that research into human protein-coding genes is disproportionately skewed towards a comparably small set of genes, and that genes that are identified by genome-wide datasets, and hence likely to have biological significance in the context of COVID-19, are at a risk of remaining ignored by researchers [11, 12]. Meta-analysis of genome-wide overlap (MAGO), or meta-analysis of over-lapping SNVs from genome-wide approaches, provides a relevant means of aggregating evidence for causal association, namely through results from meta-analysis by information content (MAIC) algorithm [13]. Notably, this study focusses the *IL-6* gene which is amongst those within the MAIC top 1000 scores (https://baillielab.net/maic/covid19/, version April 14, 2021).

Therefore, using a gene candidate approach, we aimed to analyse the impact of *IL-6* variants (rs1800797 and rs1800795) on SARS-CoV-2 infection and the clinical progression of COVID-19 in a Moroccan population.

## Materials and methods

### Patients and study design

The protocol for patient and control recruitment received approval from the ethical committee of the Faculty of Medicine of Rabat (S/22). All procedures conducted in this study adhered to the ethical standards established by the institutional and national research committee, following the principles outlined in the 1964 Helsinki Declaration and its subsequent amendments or comparable ethical standards. Written consent was obtained from all participants.

A case-control study was conducted using blood samples from unvaccinated COVID-19 patients and SARS-CoV-2-unaffected controls. The sample size was estimated using the sample size calculator server, aiming for 80% power and an expected size of 2, as previously described [14]. We enrolled 270 unvaccinated COVID-19 patients. The diagnosis of SARS-CoV-2 was established through positive real-time reverse-transcriptase-polymerase chain reaction (qRT-PCR), conducted on oro- and nasopharyngeal swab specimens, complemented by SARS-CoV-2 serology. SARS-CoV-2 infection can be grouped into the following severity of illness categories: 132 with severe COVID-19 (hospitalized for SpO2 < 94%, PaO2/FiO2 < 300 mm Hg, a respiratory rate > 30 breaths/min, or lung infiltrates > 50%), and 138 with asymptomatic to moderate COVID-19 (mild cases), (non-hospitalized), displayed fewer respiratory symptoms and did not require hospitalization for COVID-19.

Additionally, 339 SARS-CoV-2-negative group participated in routine check-up visits between December 20, 2020, and December 31, 2021, at the Pasteur Institute of Morocco, Moulay Youssef Hospital, Casablanca, and Morizgo Laboratory. The negative group consisted of participants who were PCR negative for SARS-CoV-2 RNA and negative of SARS-CoV-2 antibodies against the SARS-CoV-2 nucleocapsid protein at the time of recruitment.

### Complete blood Count (CBC)

Samples for the complete blood count were collected aseptically from each participant using a 4-mL EDTA tube (BD Microtainer, Franklin Lakes, New Jersey). The analysis was conducted using a Mindray BC-6800 analyzer (Mindray, China).

### D-dimer, C-reactive protein, and ferritin levels

D-dimer levels were assessed using the ELFA (Enzyme-Linked Fluorescent Assay) technique on a VIDAS® instrument (BioMérieux, France). C-reactive protein and ferritin were collected in a 0.5 ml serum separator tube (BD Microtainer, Franklin Lakes, New Jersey) and analyzed using a clinical chemistry analyzer (Architect ci4100, Abbott Laboratories, Abbott Park, Illinois, USA).

### SARS-CoV-2 serology

A serological assay was performed to ascertain the presence of SARS-CoV-2 antibodies against the SARS-CoV-2 nucleocapsid protein using the Architect™ SARS-CoV-2 chemiluminescence microparticles (CMIA) immunoassay (Abbott Park, Illinois, USA). The interpretation of results followed the manufacturer’s criteria: negative when the cutoff index (S/C) was < 1.4 and positive when the cutoff index (S/C) was ≥ 1.4. The SARS-CoV-2 IgG II Quant assay was utilized for the quantitative determination of IgG antibodies specific to the receptor-binding domain (RBD). This assay was conducted using the Abbott Architect i2000SR instrument manufactured by Abbott Laboratories, located in Abbott Park, Illinois.

### DNA isolation and SNPs genotyping

Genomic DNA extraction from blood was performed using the QIAamp DNA Blood Mini Kit (Qiagen, Venlo, The Netherlands). The concentration and purity of DNA were assessed using the Qubit Fluorometer (Thermo Fisher Scientific, MA, USA), following the manufacturer’s manual.

Genotyping of − 174G/C (rs1800795; assay ID C___1839697_20) and − 597 A > G (rs1800797; assay ID C___1839695_20) were conducted using predesigned TaqMan SNP genotyping assays (Thermo Fisher Scientific, MA, USA). Approximately 10 ng of genomic DNA was amplified in a 10 µL reaction mixture in a 96-well plate, containing 1x TaqMan SNP genotyping master mix (Thermo Fisher Scientific, MA, USA), SNP genotyping assay, and completed with DNase-free water. Negative controls were included in every run. The thermal profile of quantitative polymerase chain reaction (qPCR) included preincubation at 60 °C for 1 min and then 95 °C for 10 min, followed by 45 cycles of denaturation at 95 °C for 15 s, and annealing/extension at 60 °C for 1 min. This was followed by postincubation at 60 °C for 1 min and a final cooling at 40 °C for 30 s. Genotype analysis was conducted using LightCycler 480 software (Roche Diagnostics Ltd, Ferrenstrasse, Rotkreuz, Switzerland).

### Statistical analysis

For descriptive statistics, categorical variables are expressed as frequencies (%), while continuous variables are presented as the median with the minimum and maximum values. Continuous variables were compared using the Mann-Whitney U test or the Kruskal-Wallis test. Departures from Hardy-Weinberg equilibrium were assessed by comparing observed genotype frequencies with expected genotype frequencies in all groups, calculated using observed allele frequencies by the chi-square G test (‘Goodness of Fit’) with 1 degree of freedom. Odds ratios (OR) and 95% confidence intervals (CI) were estimated using logistic regression analysis and adjusted for significant covariates to examine the association between wild-type or variant genotypes among cases and controls, considering co-dominant, dominant, recessive, and overdominant inheritance models. Haplotypes frequencies were estimated by the expectation-maximization algorithm with unknown phase. The association of haplotypes with susceptibility and severity was investigated through a logistic regression model, with the most common haplotype serving as the reference. Odds ratios were adjusted for significant covariates. We conducted univariate and multivariate logistic regression analyses to explore the independent early predictors and risk factors associated with the disease severity of COVID-19. Statistical analyses were performed using R software for Windows, GraphPad Prism 6e (GraphPad Software, San Diego, CA, USA) and SNPStats (https://www.snpstats.net/start.htm) [15]. A p-value < 0.05 was considered statistically significant.

## Results

### Demographic characteristics of participants included in the study

A total of 270 unvaccinated patients with a confirmed diagnosis of COVID-19 were included in the study, consisting of 106 women and 164 men, with a median age of 52 years (range: 18–91 years). Among these, 132 patients experienced severe COVID-19 (62 women, 70 men) with a median age of 61 years (range: 21–91 years), while 138 patients had asymptomatic to moderate COVID-19 (44 women, 94 men) with a median age of 36 years (range: 18–63 years). A significant age difference was observed between severe and mild COVID-19 patients (*p* < 0.0001), with severe cases being older. Gender was also associated with the severity of COVID-19 (*p* = 0.011). Additionally, 339 SARS-CoV-2-negative group (224 women, 115 men) with a median age of 50 years (range: 19–93 years) were included.

### Analysis of single nucleotide polymorphisms in the *IL-6* gene and susceptibility to SARS-CoV-2 infection

The genotypic and allele frequencies of two SNPs in the *IL-6* gene (− 597 A > G, − 174G > C) were analyzed in 270 SARS-CoV-2-infected patients and 339 uninfected SARS-CoV-2 group, adjusting for age and sex through multivariate logistic regression analyses (Table 1). Genotypic distributions of both variants were in Hardy-Weinberg equilibrium for SARS-CoV-2-negative group (*p* = 0.28 for rs1800795 and *p* = 0.25 for rs1800797) and cases (*p* = 0.13 for rs1800795 and *p* = 0.20 for rs1800797). No significant associations were found between the allele and genotype frequencies of the *IL-6*− 597 A > G and − 174G > C variants and susceptibility to SARS-CoV-2 infection (*p* > 0.05).

**Table 1:** Genotype and allelic frequencies of two *IL–6* promoter polymorphisms among SARS-CoV-2 cases and uninfected- controls and associations with risk of COVID-19

	SARS-CoV-2 negative group (*N* = 339)	COVID-19 patients (*N* = 270)	OR [95% CI]^a^	*P*-Value
**rs1800795** Codominant				
G/G	249 (73.5)	204 (75.5)	Reference	
G/C	80 (23.6)	58 (21.5)	0.94 [0.63–1.41]	0.95
C/C	10 (2.9)	8 (3.0)	0.90 [0.34–2.41]	
Recessive model, n (%)				
G/G + G/C	329 (97.0)	262 (97.0)	Reference	–
C/C	10 (3.0)	8 (3.0)	0.91 [0.34–2.43]	0.85
Dominant model, n (%)				
G/G	249 (73.4)	204 (75.6)	Reference	–
G/C + C/C	90 (26.6)	66 (24.4)	0.94 [0.64–1.38]	0.75
Overdominant model, n (%)				
G/G + C/C	259 (76.4)	212 (78.5)	Reference	
G/C	80 (23.6)	58 (21.5)	0.95 [0.63–1.42]	0.79
Alleles frequencies ± SD				
G	0.85 ± 0.01	0.86 ± 0.02	Reference	–
C	0.15 ± 0.01	0.14 ± 0.02	0.92 [0.66–1.27]	0.60
**rs1800797**				
G/G	255 (75.2)	203 (75.2)	Reference	–
A/G	75 (22.1)	59 (21.8)	1.09 [0.73–1.64]	0.86
A/A	9 (2.7)	8 (3.0)	0.84 [0.31–2.31]	
Recessive model, n (%)				
A/G + G/G	330 (97.3)	262 (97.0)	Reference	–
A/A	9 (2.7)	8 (3.0)	0.83 [0.30–2.26]	0.71
Dominant model, n (%)				
G/G	255 (75.2)	203 (75.2)	Reference	–
A/A + A/G	84 (24.8)	67 (24.8)	1.06 [0.72–1.56]	0.78
Overdominant model, n (%)				
G/G + A/A	264 (77.9)	211 (78.2)	Reference	
A/G	75 (22.1)	59 (21.8)	1.10 [0.73–1.64]	0.66
Alleles frequencies ± SD				
G	0.86 ± 0.01	0.86 ± 0.02	Reference	–
A	0.14 ± 0.01	0.14 ± 0.02	1.02 [0.73–1.41]	0.93

^a^ OR and 95% CI were evaluated using logistic regression, adjusted for age and sex

However, a subsequent analysis revealed significant linkage disequilibrium between rs1800797 − 597 A > G) and rs1800795 (− 174G > C), suggesting that individuals with the GC* haplotype (OR = 0.04, 95%CI 0.01–0.30, *p* = 0.001 and AG* haplotype (OR = 0.11, 95%CI 0.03–0.46, *p* = 0.002) were significantly associated with protection against SARS-CoV-2 infection (Table 2).

**Table 2:** Association of *IL–6* promoter haplotypes with risk of SARS-CoV-2 infection

Haplotype	SARS-CoV-2 negative group (%)	COVID-19 patients (%)	OR [95% CI]	*P*-Value
G–G	80.6	85.9	Reference	
A–C	9.0	13.5	1.28 [0.90–2.49]	0.18
G–C	5.7	0.2	0.04 [0.01–0.30]	0.001
A–G	4.7	0.4	0.11 [0.03–0.46]	0.002

^a^ OR and 95% CI were evaluated using logistic regression, adjusted for age and sex

### Analysis of single nucleotide polymorphisms in the *IL-6* gene and susceptibility to severe COVID-19

To investigate the influence of variants in the promoter region of the *IL-6* gene on the severity of COVID-19, we genotyped the − 597 A > G and − 174G > C polymorphisms in 132 patients with severe COVID-19 and 138 asymptomatic-moderate patients (mild cases) (Table 3). The genotype distributions for both SNPs were in Hardy-Weinberg equilibrium in the mild group (*p* = 1.00 for both rs1800795 and rs1800797 SNPs) but not in the severe COVID-19 cases (*p* = 0.01 for rs1800795 and *p* = 0.02 for rs1800797).

**Table 3:** Allele frequencies of two *IL-6* polymorphisms among COVID-19 patients with severe and mild symptoms

	Mild cases (*N* = 138)	Severe cases (*N* = 132)	OR [95% CI]^a^	*P*-Value
**rs1800795** Codominant				
G/G	101 (73.2)	103 (78.0)	Reference	
G/C	35 (25.4)	23 (17.4)	0.42 [0.18–0.98]	0.09
C/C	2 (1.4)	6 (4.6)	2.14 [0.20–22.93]	
Recessive model, n (%)				
G/G + G/C	136 (98.5)	126 (95.5)	Reference	–
C/C	2 (1.5)	6 (4.5)	2.56 [0.24–26.87]	0.41
Dominant model, n (%)				
G/G	101 (73.2)	103 (78.0)	Reference	–
G/C + C/C	37 (26.8)	29 (22.0)	0.51 [0.23–1.13]	0.10
Overdominant model, n (%)				
G/G + C/C	103 (74.6)	109 (82.6)	Reference	
G/C	35 (25.4)	23 (17.4)	0.41 [0.18–0.96]	0.03
Alleles frequencies ± SD				
G	0.86 ± 0.02	0.87 ± 0.02	Reference	–
C	0.14 ± 0.02	0.13 ± 0.02	0.93 [0.57–1.52]	0.77
**rs1800797** Codominant				
G/G	101 (73.2)	102 (77.3)	Reference	–
A/G	35 (25.4)	24 (18.2)	0.44 [0.19–1.04]	0.12
A/A	2 (1.4)	6 (4.5)	2.17 [0.20–23.21]	
Recessive model, n (%)				
A/G + G/G	136 (98.5)	126 (95.5)	Reference	–
A/A	2 (1.5)	6 (4.5)	2.56 [0.24–26.87]	0.41
Dominant model, n (%)				
GG	101 (73.2)	102 (77.3)	Reference	–
A/A + A/G	37 (26.8)	30 (22.7)	0.53 [0.24–1.19]	0.12
Overdominant model, n (%)				
G/G + A/A	103 (74.6)	108 (81.8)	Reference	
A/G	35 (25.4)	24 (18.2)	0.43 [0.19–1.01]	0.05
Alleles frequencies ± SD				
G	0.86 ± 0.02	0.86 ± 0.02	Reference	–
A	0.14 ± 0.02	0.14 ± 0.02	0.96 [0.59–1.56]	0.87

^a^ OR and 95% CI were evaluated using logistic regression, adjusted for Age and sex

In the overdominant model, the frequency of the G/C genotype of the rs1800795 polymorphism (− 174G > C) was significantly higher in mild cases than in severe patients (*p* = 0.03). Individuals with the G/C genotype were protected against progression to severe disease compared with those with the G/G or C/C genotypes (*p* = 0.03; OR = 0.41, 95%CI 0.18–0.96) (Table 3). After the Bonferroni correction (adjusted alpha = 0.005), we observed no significant difference in the rs1800795 polymorphism (− 174G > C) between severe and mild cases.

Regarding the rs1800797 (− 597 A > G) polymorphism, there was no significant statistical association between different genotypes and severity under various genetic models (*p* > 0.05). Haplotype analysis of the two *IL-6* gene polymorphism loci (− 597 A > G and − 174G > C) was performed, as shown in Table 4. The probability of developing a severe form of COVID-19 was not linked to any haplotype (*p* > 0.05).

**Table 4:** Association between COVID-19 severity and major haplotypes of rs1800797 and rs1800795

Haplotype	Mild cases (%)	Severe cases (%)	OR [95% CI]	*P*-Value	
G–G	85.5	86.4	Reference		
A–C _*_–_*_	13.8 0.7	13.3 0.3	0.70 [0.35–1.37] 0.23 [0.01–4.15]	0.30 0.32	

_*_–_* rare_

^a^ OR and 95% CI were evaluated using logistic regression, adjusted for age and sex

Multivariate analysis indicated that the age of patients (OR = 1.06; 95% CI, 1.04–1.08, < 0.001), D-dimer (OR = 1.40; 95% CI, 1.06–1.56, *p* = 0.002), and CRP levels on admission (OR = 1.12; 95% CI, 1.06–1.26, *p* = 0.003) were risk factors associated with severe COVID-19 (Supplementary Table 1).

### *IL-6* gene polymorphisms and biological markers

**Fig. 1:**
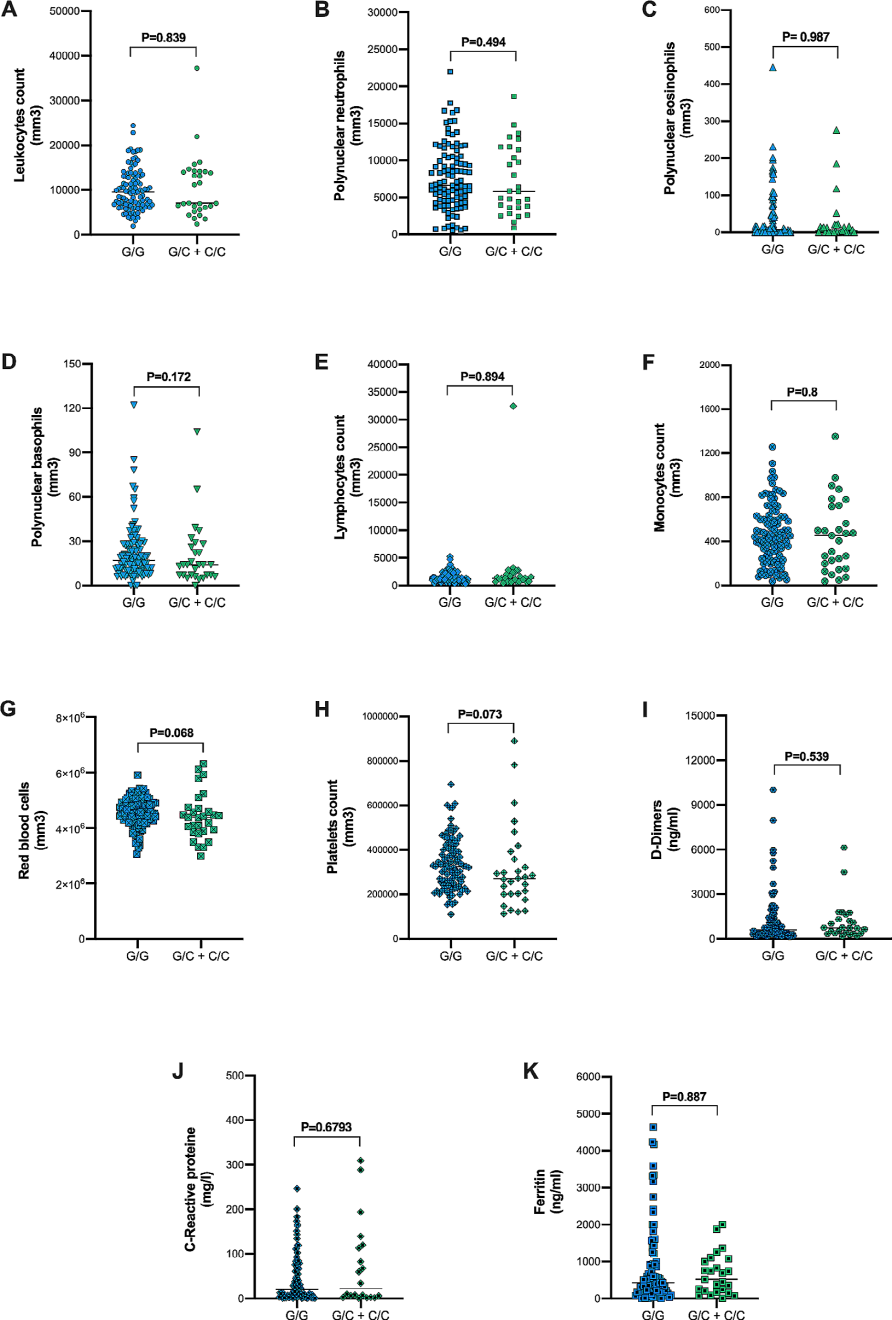
Biological markers in the COVID-19 cases according to *IL-6* rs1800795 variant. **A**) *IL-6* rs1800795 genotypes and leukocytes count. **B**) *IL-6* rs1800795 genotypes and polynuclear neutrophils count. **C**) *IL-6* rs1800795 genotypes and polynuclear eosinophils count. **D**) *IL-6* rs1800795 genotypes and polynuclear basophils count. **E**) *IL-6* rs1800795 genotypes and lymphocytes count. **F**) *IL-6* rs1800795 genotypes and monocytes count. **G**) *IL-6* rs1800795 genotypes and red blood cells count. **H**) *IL-6* rs1800795 genotypes and platelets count. **I**) *IL-6* rs1800795 genotypes and D-dimers count. **J**) *IL-6* rs1800795 genotypes and C-reactive protein levels. **K**) *IL-6* rs1800795 genotypes and ferritin levels. Data are presented as a scatter plot with median. Statistical tests were performed using the Mann Whitney U test

**Fig. 2:**
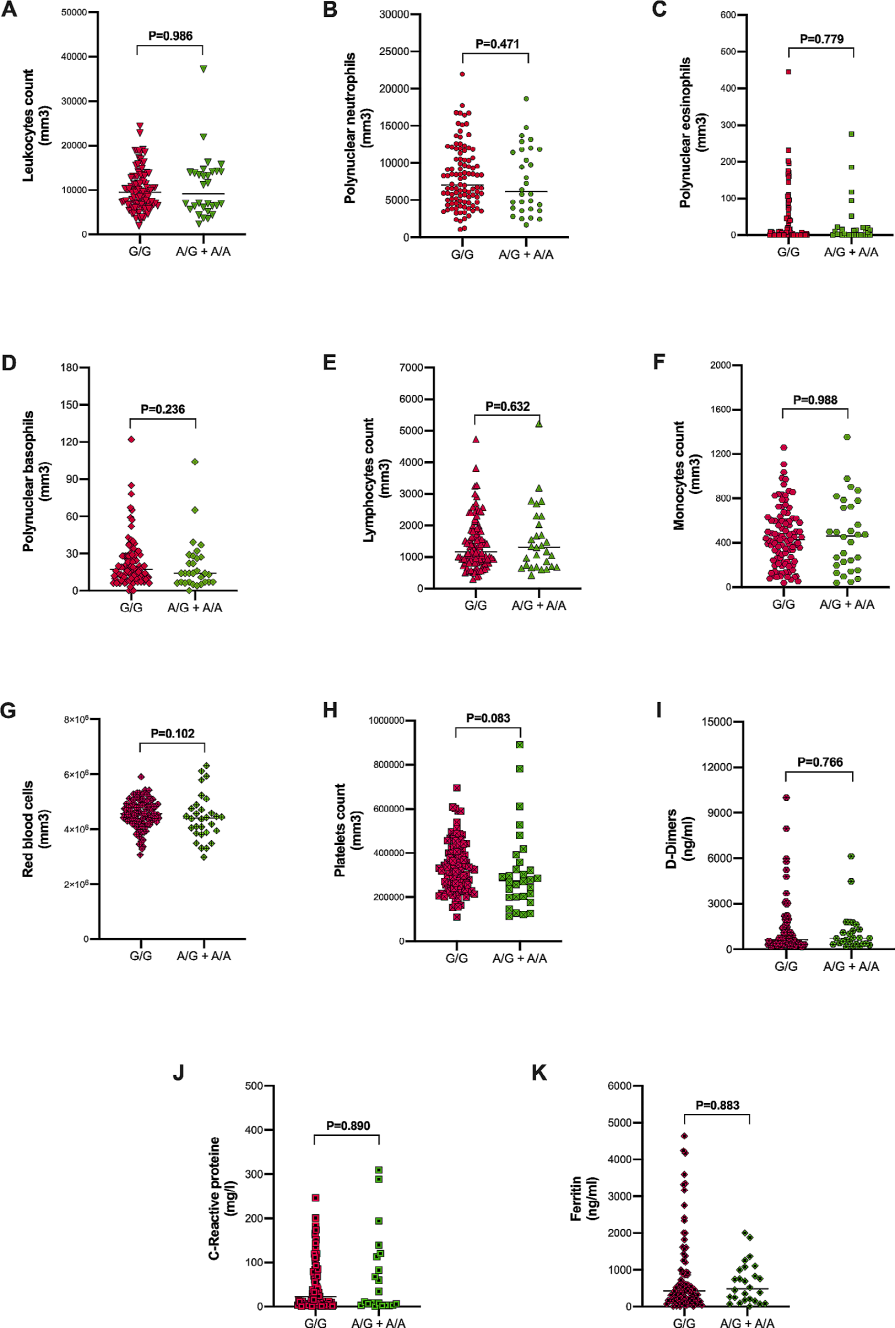
Biological markers in the COVID-19 cases according to *IL-6* rs180097 polymorphism. **A**) *IL-6* rs180097 genotypes and leukocytes count. **B**) *IL-6* rs180097 genotypes and polynuclear neutrophils count. **C**) *IL-6* rs180097 genotypes and polynuclear eosinophils count. **D**) *IL-6* rs180097 genotypes and polynuclear basophils count. **E**) *IL-6* rs180097 genotypes and lymphocytes count. **F**) *IL-6* rs180097 genotypes and monocytes count. **G**) *IL-6* rs180097 genotypes and red blood cells count. **H**) *IL-6* rs180097 genotypes and platelets count. **I**) *IL-6* rs180097 genotypes and D-dimers count. **J**) *IL-6* rs180097 genotypes and C-reactive protein levels. **K**) *IL-6* rs180097 genotypes and ferritin levels. Data are presented as a scatter plot with median. Statistical tests were performed using the Mann Whitney U test

We conducted an analysis to explore the potential impact of *IL-6* polymorphisms, specifically rs1800795 and rs1800797, on complete blood count (CBC) and D-dimer levels in COVID-19 patients. The results are depicted in Figs. 1 and 2 for rs1800795 and rs1800797, respectively. Our findings revealed no significant differences between genotypes and the levels of hematological markers, C-reactive protein, and ferritin levels, based on the − 597 A > G and − 174G > C genotypes.

## Discussion

The clinical presentation of COVID-19 varies among patients. According to early Moroccan data, 64.4% of patients developed non-severe symptoms, while 33.6% were admitted to the intensive care unit [16]. An individual’s genetic background may influence the outcome of SARS-CoV-2 infection. Variability in susceptibility to SARS-CoV-2 and the severity of COVID-19 involves multiple factors, encompassing both viral and host elements, with genetic factors playing a role in affecting proinflammatory cascades [17]. IL-6, a pro-inflammatory cytokine, plays a crucial role in the immune response and inflammation. Previous suggestions have implicated its involvement in the severity of COVID-19, with elevated IL-6 levels being associated with a more severe disease course [18, 19]. Understanding the genetic factors that influence the severity of COVID-19 disease has clinical implications. Identifying individuals with potentially protective genotypes can contribute to risk stratification and the development of personalised treatment strategies. In our study, a significant age difference was observed between severe and mild COVID-19 patients, supporting the well-established finding that age plays an important role in disease severity [20–23]. Furthermore, the association between male gender and the severity of COVID-19 is consistent with previous observations. These findings underscore that sex hormones, angiotensin-converting enzyme levels, and severe inflammation in men may contribute to the progression from mild to severe cases [20, 24–26].

Earlier data revealed a systemic pro-inflammatory signature, in particular elevated levels of IL-6, which were associated with both severity of organ failure and 60-day mortality [27]. In this study, we explored the association between *IL-6* polymorphisms (− 174G > C and − 597 A > G) and susceptibility to SARS-CoV-2 infection. However, allele and genotype frequencies revealed no significant association. These results are consistent with those of a study conducted in the Chinese population, which also reported non-significant associations in allele and genotype frequencies between healthy controls and COVID-19 patients [28].

Interestingly, our study showed interesting results when haplotypes were taken into account. Linkage disequilibrium analysis showed that individuals with specific haplotypes (GC* and AG*) were significantly associated with protection against SARS-CoV-2 infection. This finding suggests that certain combinations of alleles may confer a protective effect, potentially influencing the host response to the virus. This observation is in line with previous in vitro studies indicating that genetic polymorphisms in the *IL-6* gene promoter influence its transcription by a complex haplotype-determined interaction, rather than by a simple additive mechanism [9]. Polymorphisms, such as the − 597 A > G and − 174G > C SNPs, located adjacent to the negative regulatory domain (nucleotides − 173 to − 151 relative to the transcription start site) and in close proximity to an NF-IL6 binding motif, appear to contribute to interindividual variation in IL-6 transcription and expression [28]. Additionally, in an ex vivo model, *IL-6* promoter haplotypes defined by the − 597 and − 174 SNPs are associated with IL-6 production stimulated by endotoxin [28]. An early Turkish study found that the GG genotype of the − 174G > C polymorphism was significantly higher in COVID-19 patients with macrophagic activation syndrome. The G allele may serve as a risk factor for elevated serum IL-6 levels and progression to macrophagic activation syndrome [29]. An investigation conducted in Iraq supports the concept that the *IL-6*− 597 A > G variant is associated with susceptibility to HBV and HCV infections [30]. However, a Chinese study found no association between HBV resistance and the − 174G > C and − 597 A > G variants [31]. Interestingly, among HBV patients, the AG* haplotype was significantly associated with an increased risk of developing HBV infection (OR = 5.45; 95% CI: 3.63–8.18; *p* = 0.000), whereas a protective effect of the GC* haplotype against the progression of HBV infection was suggested. For HCV, the AC* haplotype was significantly associated with the risk of HCV infection (OR = 1.96, 95% CI: 1.31–2.93; *p* = 0.001) [30].

Under the overdominant model analysis, we demonstrated that the IL-6 − 174 G/C genotype confers protection against the development of severe COVID-19 after adjusting for sex and age. Consistent with a previous Chinese study, it was identified that rs1800796 C/C and rs1524107 T/T protect against severe COVID-19 [28]. Consistent with a previous meta-analysis, it was also shown that the C allele of the − 174G > C SNP is linked to higher IL-6 production and more severe COVID-19 in the Caucasian population [32]. Furthermore, a previous study demonstrated a high correlation between the C allele and elevated expression of IL-6 compared to the ancestral allele (G) [28]. In contrast to our study, an Indian report showed that individuals with the G/C genotype of the IL-6 (–174G > C) polymorphism have a significantly higher risk of severity (adjusted OR = 3.86, *p* < 0.001) [33].

For the rs1800797 (− 597 A > G) polymorphism, we did not find any association with the severity of COVID-19. Previous data from an Indian study [33] and the Iranian population [34] align with our findings and showed no association of COVID-19 severity with the rs1800797 (− 597 A > G) SNP [33].

Infection with SARS-CoV-2 induces an inflammatory response, resulting in a significant release of pro-inflammatory cytokines, leading to a cytokine storm. Numerous studies have investigated the cytokine profiles of individuals with COVID-19, revealing a strong correlation between the cytokine storm and a poor prognosis in severe cases of COVID-19 [4].

Furthermore, the probability of developing a severe form of COVID-19 was not linked to any haplotype. However, an Asian study showed that the common CTT* haplotype, represented by the rs1800796, rs1524107 and rs2066992 alleles, contributed to a better outcome from SARS-CoV-2 infection and was associated with a reduction in IL-6 expression [28].

These differences in results could be attributed to ethnic differences, as the viruses are also genetically distinct. The underlying immunopathogenesis of viral infections is thought to involve different mechanisms, influencing the outcome [35].

The absence of a statistically significant association between these IL-6 genotypes and the levels of complete blood count, D-dimer, CRP, and ferritin levels is consistent with findings from previous studies in other inflammatory contexts [36–39].

It’s important to acknowledge the limitations of the study, such as the relatively small sample size and potential confounding factors. Future research involving genome-wide association study (GWAS) with larger and more diverse cohorts and ex vivo functional studies could further validate these findings. Additionally, more attention should be paid to evaluating IL-6 serum levels in patients with GC* and AG* haplotypes in order to establish the genotype-to-phenotype relationship.

In conclusion, the GC* and AG* haplotypes of rs1800795 (− 174 G > C) and rs1800797 (− 597 A > G) variants in the *IL-6* gene may serve as useful genetic markers to screen for high-risk SARS-CoV-2 infection in the Moroccan population, providing valuable insights into the genetic determinants of COVID-19 severity. The study’s results may have implications for public health strategies, such as identifying high-risk populations based on genetic factors and tailoring preventive measures accordingly. Further study is warranted to confirm these findings in larger cohorts and diverse populations, paving the way for more targeted approaches in managing and predicting severe COVID-19 cases.

### Electronic supplementary material

Below is the link to the electronic supplementary material.

Supplementary Material 1

## Data Availability

The data presented in this study are available upon reseanable request from the corresponding author. The data are not publicly available according to the ethical committee decision on the conduct of this study.
