# Supplementary material for: The Interleukin-6 gene variants may protect against SARS-CoV-2 infection and the severity of COVID-19: a case-control study in a Moroccan population
Source: BMC Med Genomics. 2024 May 23;17:139. doi: 10.1186/s12920-024-01911-w (PMC11112821; doi:10.1186/s12920-024-01911-w)
Supplement: Supplementary file 1 — Supplementary Material 1 [file 12920_2024_1911_MOESM1_ESM.docx]

| **Variables** | **Univariate analysis** | | **Multivariable analysis** | |
| --- | --- | --- | --- | --- |
|  | OR [95 % CI] | p-value | OR [95 % CI] | p-value |
| Age  Sex (female)  D-dimer  CRP  IgG anti-N  IgG anti-RBD  ***Il-6* rs180095 CC/GC**  ***Il-6* rs180097 AA /AG** | 1.06 [1.04–1.08]  1.37 [0.90–2.09]  1.60 [1.02–1.30]  1.14 [1.07–1.20]  1.02 [0.94–1.11]  1.10 [1.01–1.13]  **0.94 [0.64–1.38]**  **1.06 [0.72–1.56]** | <0.001  0.14  <0.001  <0.001  0.57  0.001  **0.75**  **0.78** | 1.06 [1.04–1.08]  -  1.40 [1.06–1.56]  1.12 [1.06–1.26]  -  1.08 [1.00–1.10]  -  - | <0.001  -  0.002  0.003  -  0.09  -  - |

**Supplementary table 1**: Protective factors against COVID-19 disease progression comparing patients with mild SARS-CoV-2 infection with severe patients
